# The impact of digital healthcare systems on pain and body function in patients with knee joint pain: a systematic review and meta-analysis

**DOI:** 10.1038/s41598-024-53853-z

**Published:** 2024-02-09

**Authors:** Longfei Guo, Shuoqi Li, Shihao Xie, Lin Bian, Shazlin Shaharudin

**Affiliations:** 1 School of Health Sciences, Universiti Sains Malaysia, Kota Bharu, Kelantan Malaysia; 2 School of Sports Science, Nantong University, Nantong, Jiangsu China; 3 School of Physical Education, Woosuk University, Jeonju, Korea

**Keywords:** Pain, Health care, Bone, Cartilage, Muscle, Skeleton

## Abstract

The digital healthcare (DH) system has recently emerged as an advanced rehabilitation approach that promotes rehabilitation training based on virtual reality (VR) and augmented reality (AR). The purpose of this meta-analysis study is to review and assess the impact of DH systems on pain and physical function among patients diagnosed with knee joint pain. Between January 2003 and September 2023, studies that met the listed inclusion criteria were gathered from Scopus, PubMed, Web of Science, and EBSCO databases. The analysis of standardized mean difference (SMD) was carried out with 95% confidence interval (95% CI) (PROSPERO registration number: CRD42023462538). Nine research papers were selected, which collectively involved 194 males and 279 females. The meta-analysis outcomes revealed that DH intervention significantly improved balance (SMD, 0.41 [0.12, 0.69], p < 0.05) and pain level (SMD, − 1.10 [− 2.02, − 0.18], p < 0.05). The subgroup analysis of the pain level showed varied outcomes for the TKA (SMD, − 0.22 [− 0.49, 0.04], p = 0.10) or OA patients (SMD, − 2.80 [− 3.83, − 1.78], p < 0.05) Next, this study found no significant effect of DH intervention on knee joint range of motion (ROM) (SMD, 0.00 [− 0.76, 0.76], p = 1.00) and walking velocity (SMD, 0.04 [− 0.22, 0.29], p = 0.77) in patients with knee joint pain. The meta-analysis review conducted in this study revealed that DH intervention may potentially improve balance among the patients with knee joint pain. It may also alleviate the pain level particularly among OA patients.

## Introduction

Total knee arthroplasty (TKA) and knee osteoarthritis (OA) are the main causes of knee joint pain. Besides limiting knee movement, these knee-related issues cause atrophy of muscles around the knee joint and severely affect the function of knee joint^1^. Knee injuries can potentially trigger serious fibrous exudate and fibrin in the interstitial space deposited in the knee joint cavity, which can further lead to fibrous adhesion^2^. Long-term immobilization due to knee pain induces osteoporosis, causes muscle atrophy, articular cartilage nutritional disorder, and fibrosis^3–5^. In addition, joint cavity stenosis may occur as the synovial sacs dry up stemming from prolonged immobilization of the knee joints^6^.

The digital healthcare (DH) system has recently emerged as an advanced rehabilitation approach that promotes rehabilitation training based on virtual reality (VR) and augmented reality (AR). In VR games, real-like worlds are simulated by using interactive computer settings to improve the daily activities of patients or their functional movements^7^. This is achievable by deploying AR technology that superimpose computer-generated virtual objects on real images to yield real-world simulations^8^. The patients are given a unique avatar that represents their body movements^7^ to interact with the virtual environment by using specific devices or with body movements. Apart from offering a sense of reality, telerehabilitation integrated with AR enhances postural perception by enabling simultaneous real-time interaction with virtual objects (in the virtual world) and physical settings (in the real world)^9^.

Prior studies^10,11^ revealed that DH systems effectively improved pain and body function recovery in patients suffering from knee joint pain when compared to standard rehabilitation (SR) techniques. For example, Nambi et al.^11^ compared the pain level and physical function of the knee among OA patients subjected to 4 weeks of VR games training and SR training, with 20-min five sessions per week. The study disclosed significant improvement in knee joint movements and pain levels after implementing VR training when compared to the outcomes derived from SR training. On the contrary, other studies^12,13^ reported that DH systems had no impact on TKA patients. Therefore, the efficacy of DH systems to rehabilitate knee joint pain remains debatable.

A meta-analysis study^7^ showed that VR games could alleviate pain levels in chronic neck pain and shoulder impingement syndrome, but its effect on knee joint pain was not disclosed. Therefore, this present meta-analysis study systematically reviewed and assessed the impact of DH systems on pain and physical function among patients diagnosed with knee joint pain.

## Methods

### Study selection and data collection

**Figure 1:**
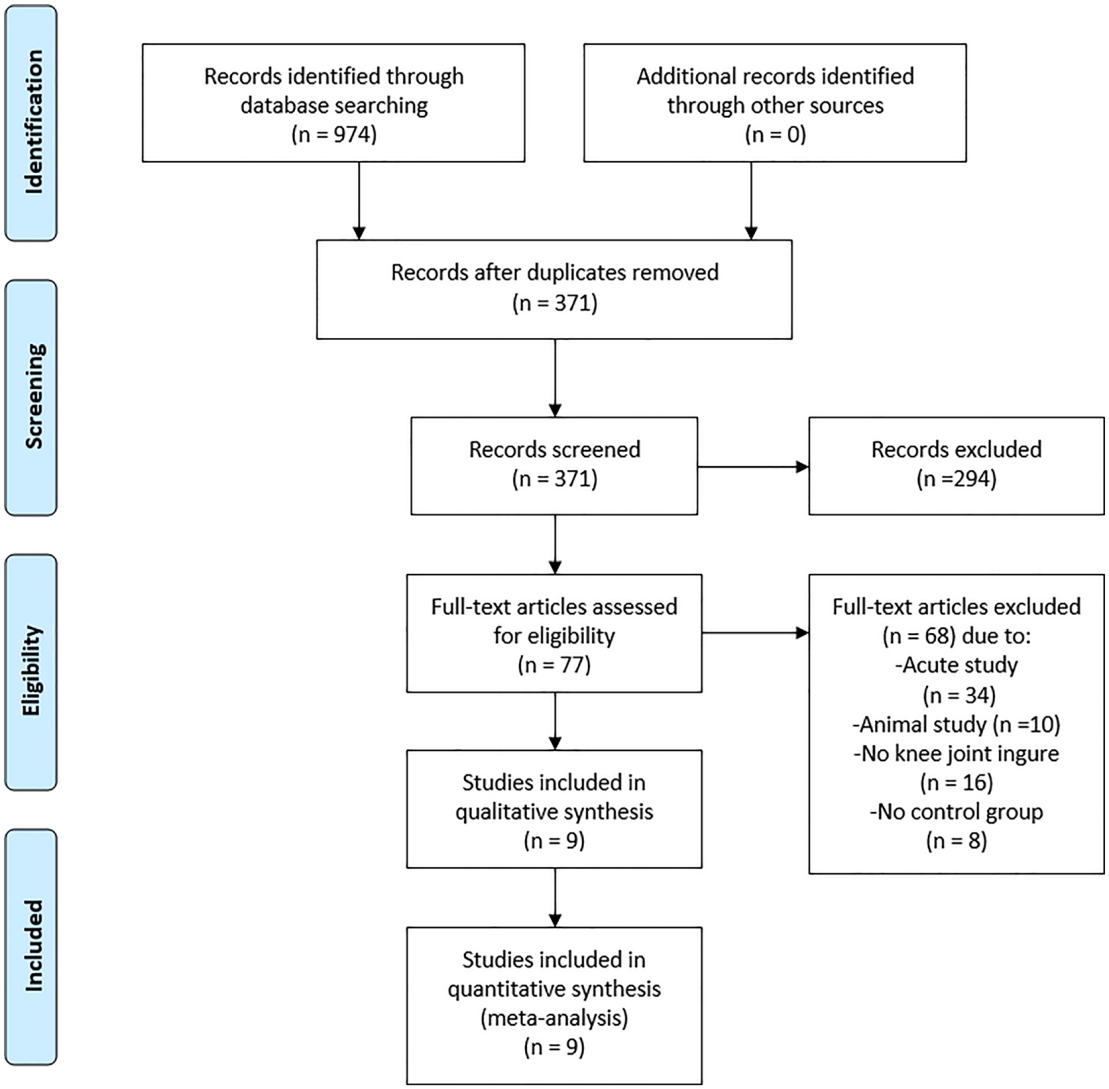
Flow diagram of the search results using the preferred reporting items for systematic reviews and meta-analysis (PRISMA).

The protocol for this meta-analysis review was registered in the PROSPERO database (CRD42023462538) on September 21, 2023. As depicted in Appendix A, two researchers were responsible for the search strategy and manuscript preparation by adhering to the Preferred Reporting Items for Systematic Reviews and Meta-Analysis (PRISMA) guidelines. The search strategy was executed by identifying articles published between January 1, 2003, and September 20, 2023, from four electronic databases, namely EBSCO, PubMed, Scopus, and Web of Science. We chose to search for studies from the past 20 years^14^ because the number of published studies on digital healthcare systems has been continuously increasing from 2003 (Pubmed: 199 studies) to 2023 (Pubmed: 4321 studies). The following keywords were used to search through the databases: “Knee”, “Training”, “Exercise”, “Virtual reality”, and “Augmented reality”. Subsequently, the screening process (title and abstract) was performed by two independent investigators. The selected full articles were re-screened based on the inclusion and exclusion criteria set for this study. The two independent investigators conducted a quality assessment and a data extraction process for articles that met the inclusion criteria. All data were retrieved from the published literature. In the event of any dispute regarding the status of an article, another independent investigator was invited to weigh in on the decision until a consensus was achieved. The respective reference was acquired manually once a study inclusion was confirmed. Figure 1 illustrates the article selection protocol deployed in this study.

### Inclusion and exclusion criteria

The article selection was performed by adhering closely to the following inclusion criteria: (1) patients with knee joint pain, (2) studies that conducted the randomized controlled trial (RCT) approach, (3) either VR or AR training was assigned to the DH group, while SR training was assigned to the SR group (examples of SR interventions: knee joint stretching, strength, and balance training), (4) pre- and post-training analyses that included pain level, balance, walking velocity (WV), and range of motion (ROM) of knee joint, (5) findings expressed in median (interquartile range) or mean ± standard deviation, (6) articles published between January 2003 and September 2023, and (7) articles written in English. The only exclusion criterion for article selection in this review refers to articles derived from grey literature.

### Quality assessment

The quality of the selected articles was determined by using the Cochrane risk of bias assessment tool^15^. The assessed quality aspects comprised of blinding of outcome assessment, participants and personnel blinding, allocation concealment, incomplete data, random sequence generation, and selective reporting^16^. Each article was given a score of “yes”, “no” or “unclear”. Two co-authors independently (L.G. and S.L.) assessed the quality of each study, whereas another author (S.X.) facilitated the decision-making upon any disagreement.

### Data extraction

Based on the previous research paradigm^17^, the content to be extracted was formulated, and the extracted content was optimized through the studies included in the initial search^10,13^. Details from each included study were extracted and tabulated, such as age, gender, disease type, DH device, training duration, index, traditional training (TR) and DH protocol (see Table 1). Data extraction was performed independently by two co-authors (L.G. and S.L.) Any disagreement was resolved by another researcher (S.X.)

**Table 1:** Characteristics of included studies.

Study	Age (y)	Gender (M/F)	Disease	DH device	Duration	TR protocol	DH protocol	Index
Gianola 2020	68.6 ± 8.8	37/48	TKA	VR	10 days; 1x/day	Passive knee motion and walking; 60 min	Similar exercises for goals with VR	Pain, ROM
Hadamus 2021	68.5 ± 6.3	14/28	TKA	VR	4 weeks; 5x/week	Knee range of motion, gait and balance, manual therapy and massage	Additionally received 3x/week of VR games	Balance
Hadamus 2022	68.5 ± 6.4	19/40	TKA	VR	4 weeks; 5x/week	Knee range of motion, gait and balance, manual therapy and massage	Additionally received 3x/week of VR games	WV
Li 2022	32.7 ± 7.7	18/22	Knee joint injury	AR	14 days; 1x/day	Active and passive ankle joint function exercises	A virtual knee joint was constructed in the exercise therapy to drive the real knee joint to carry out training	Pain
Nambi 2020	22.3 ± 1.3	40/0	Knee OA	VR	4 weeks; 5x/week	Resistance training for muscles around the knee joint, 15 repetitions, 3 sets, 20 min	VR games that focus on knee joint movements, 20 min	Pain
Ozlu 2023	53.5 ± 9.9	30/43	Knee OA	VR	3 weeks; 5x/week	Transcutaneous electrical nerve stimulation on the knee; 20 min; 100 MHz	Additionally received 3x/week of VR games	Pain, balance, WV
Pournajaf 2020	69.7 ± 6.9	22/34	TKA	VR	3 weeks; 5x/week	Balance and proprioception training; 45 min	Similar exercises for goals with VR	Pain, WV
Shim 2023	70.6 ± 6.6	11/43	TKA	AR	12 weeks; 7x/week	ROM, gait, balance and strengthening exercise	Lower limb range of motion, stretching and isometric exercises based on AR	Pain, ROM, balance, WV
Yu 2023	68.9 ± 3.7	3/21	TKA	AR	4 weeks; 3x/week	Passive range of motion and continuous passive motion training; 30 min	Lower limb function exercise based on AR	Pain, ROM, balance

*M* male, *F* female, *TKA* total knee arthroplasty, *OA* osteoarthritis, *VR* virtual reality, *AR* augmented reality, *ROM* range of motion, *WV* walking velocity.

### Data analysis

The meta-analysis was performed by using the Review Manager (RevMan) software (version 5.4.1, Copenhagen: The Nordic Cochrane Centre, The Cochrane Collaboration, 2020) to assess the impact of the interventions. The standardized mean difference (SMD) was selected as the most suitable effect scale index after considering that the reviewed articles recorded continuous variable outputs with varying test methods. Next, median (range) data were standardized by converting them into mean ± standard deviation^18^. In cases where data were not disclosed, the authors of the respective articles were contacted by email to gather clarifications.

When more than five studies displayed a similar indicator, the sensitivity analysis was carried out by excluding each study in sequence to assess the stability of the meta-analysis outcomes. Study heterogeneity was applied to examine I^2^ statistics. A small I^2^ indicates low heterogeneity between studies, whereas an I^2^ below 50% implies homogeneous studies. In the current review, a fixed effect model was applied for data analysis. On the contrary, I^2^ ≥ 50% suggests heterogeneous studies, thus the random effect model will be applied for data analysis^19^. The publication bias was evaluated by using funnel plot, while the SMD was evaluated by using the Forest plot. Finally, the uncertainty level was calculated at 95% CI.

## Results

### Eligibility of studies

The systematic review was conducted based on nine RCT articles that met the inclusion criteria. Each included study indicated ethical approval of the study protocol from the respective institutions. Two independent co-authors reported a high consistency level with each other (Cohen kappa coefficient = 0.89) during the screening process. Out of the 473 patients from the included articles, 194 were males and 279 were females. As for group division, 246 and 227 patients were assigned to DH and SR groups, respectively. Six studies used VR tools for DH training, while three studies used AR technology. The shortest intervention time was 10 days and the longest was 12 weeks.

### Quality assessment

**Figure 2:**
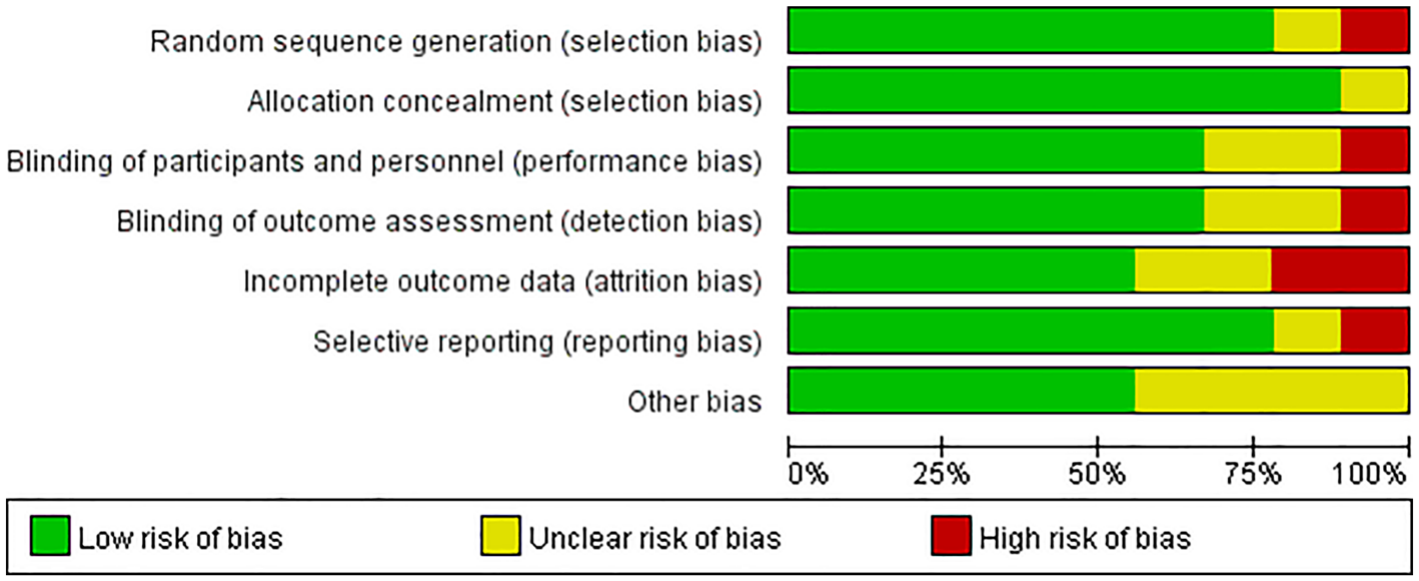
Analysis of risk of bias according to Cochrane Collaboration guideline.

The overall quality of the nine articles was relatively high, with most of the articles displaying a low risk of bias (69.9%) (Fig. 2). Meanwhile, a small percentage of the articles were highly biased (9.5%) and the remaining articles appeared to be unclear (20.6%).

### Quantitative synthesis

**Figure 3:**
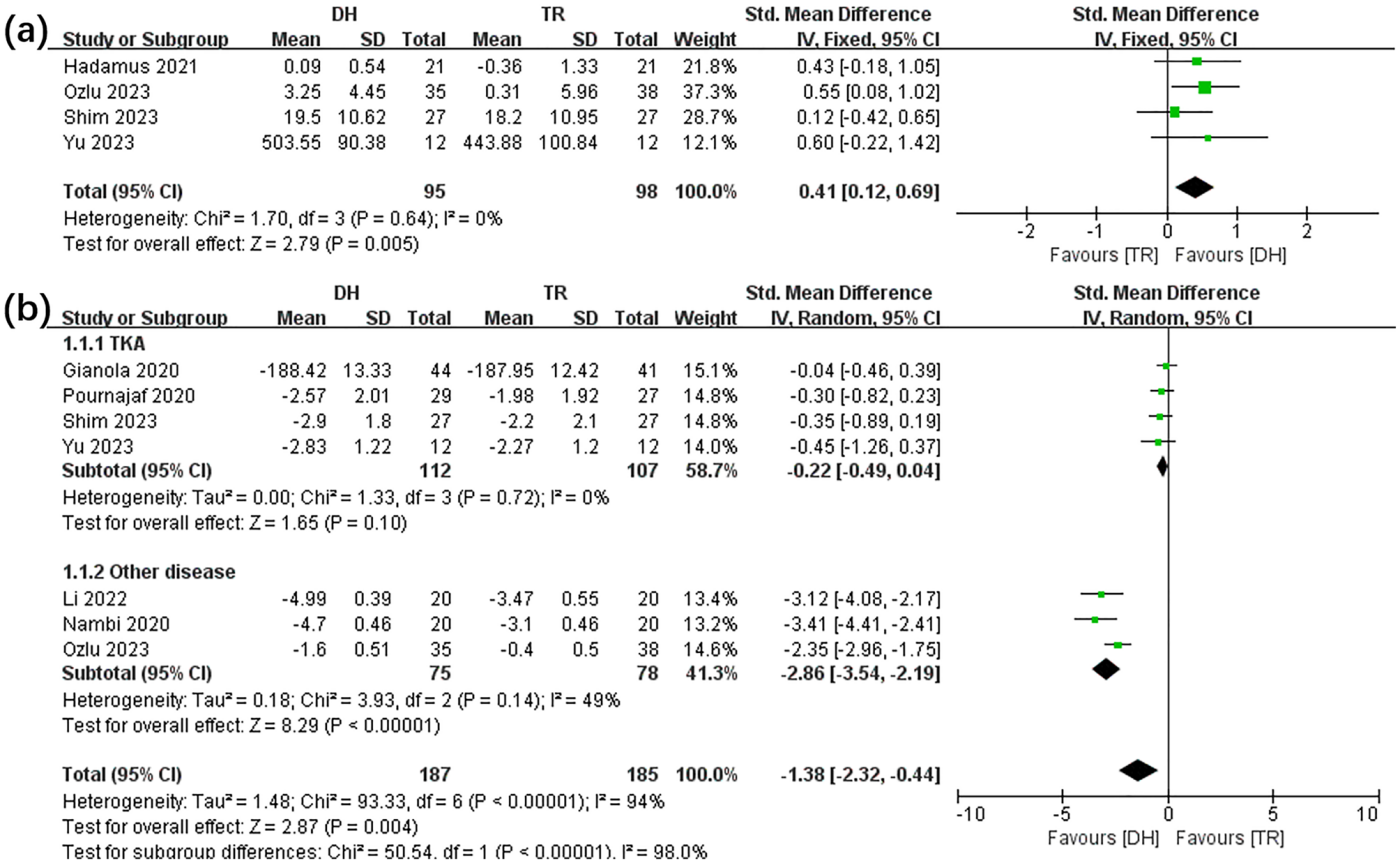
Forest plot illustrates the effects of TR versus DH intervention on balance (**a**) and pain level (**b**).

The effects of DH and SR groups on balance were compared in four^10,13,20,21^ included articles (see Fig. 3a) while the pain level (see Fig. 3b) were compared in seven^10–13,21–23^ included articles. Apparently, the meta-analysis for balance (SMD, 0.41 [0.12, 0.69], p < 0.05, I^2^ = 0%, p for heterogeneity = 0.64) and pain level (SMD, − 1.10 [− 2.02, − 0.18], p < 0.05, I^2^ = 95.6%, p for heterogeneity < 0.05) showed that enhanced balance was more prevalent in the DH group than in the SR group. The subgroup analysis of the pain level showed varied outcomes for the TKA (SMD, − 0.22 [− 0.49, 0.04], p = 0.10, I^2^ = 0%, p for heterogeneity = 0.72) and OA (SMD, − 2.80 [− 3.83, − 1.78], p < 0.05, I^2^ = 68%, p for heterogeneity = 0.08) patients.

**Figure 4:**
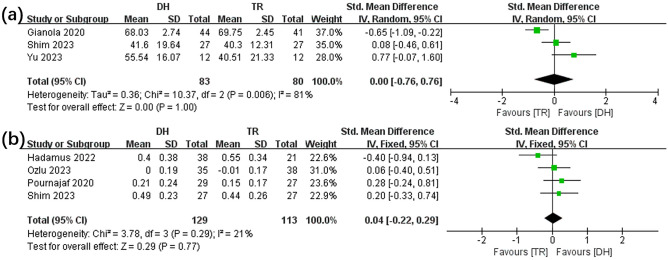
Forest plot illustrates the effects of TR versus DH intervention on ROM (**a**) and WV (**b**).

The effects of DH and SR groups on ROM were compared in three^13,21,22^ included articles (see Fig. 4a), while the WV (see Fig. 4b) were compared in four^10,12,13,24^ included articles. Interestingly, the meta-analysis detected an insignificant variance in the ROM (SMD, 0.00 [− 0.76, 0.76], p = 1.00, I^2^ = 81%, p for heterogeneity < 0.05) and WV (SMD, 0.04 [− 0.22, 0.29], p = 0.77, I^2^ = 21%, p for heterogeneity = 0.29) between the DH and SR groups.

### Publication bias analysis

**Figure 5:**
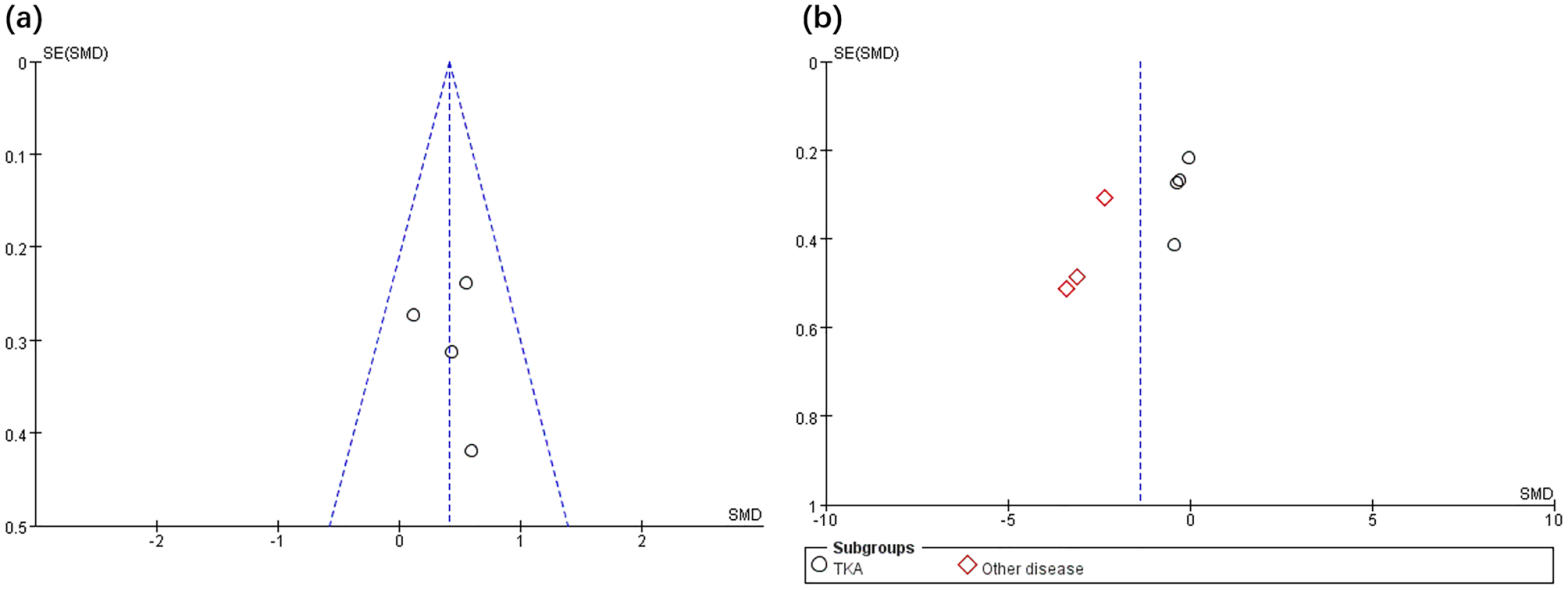
Funnel plot of publication bias for balance (**a**) and pain level (**b**) in the TR versus DH intervention.

**Figure 6:**
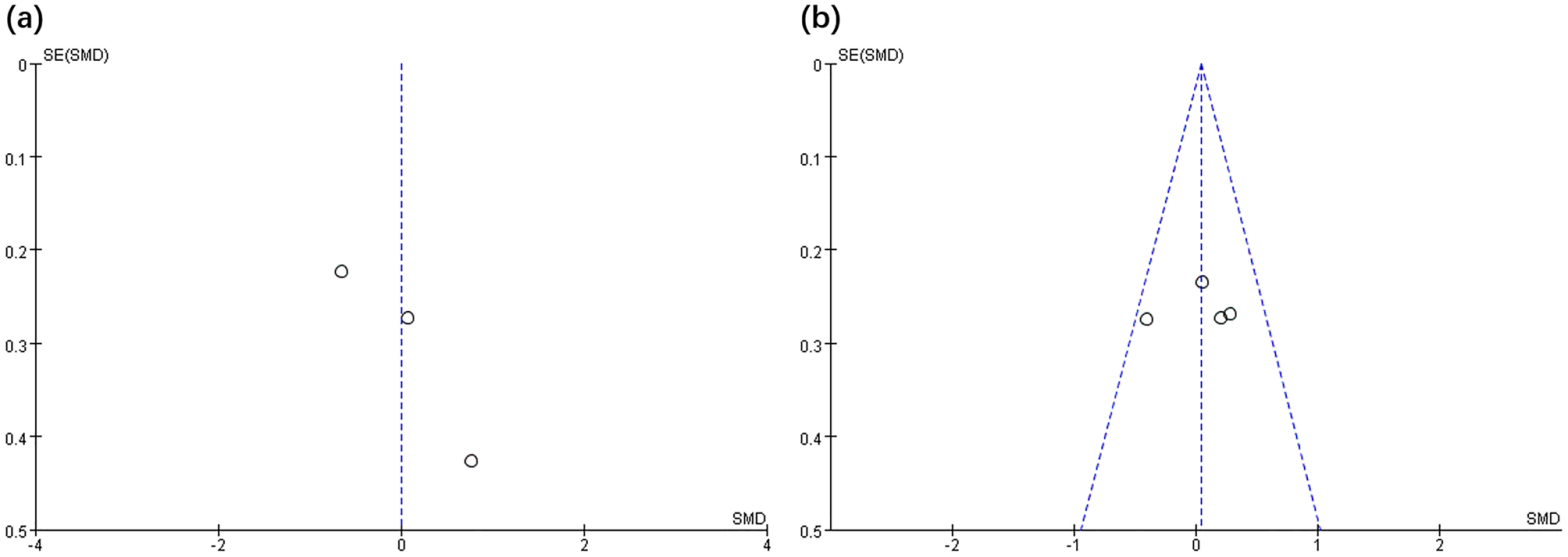
Funnel plot of publication bias for ROM (**a**) and WV (**b**) in the TR versus DH intervention.

This review includes 9 studies, which is close to the minimum requirement for using funnel plots. Publication bias can be reflected to some extent, and the presence of small sample publication bias has also been noted in previous studies^14,16,25^. As illustrated in Figs. 5 and 6, the publication bias analysis showed that the analysis yielded a left–right symmetrical distribution that indicated a low probability of publication bias.

### Sensitivity analysis

The meta-analysis revealed no significant changes in each group after the analysis type was modified, the impact size was changed, and the individual studies were excluded. Thus, the sensitivity analysis showed that the research results were reliable.

## Discussion

This study assessed the impact of DH systems on balance, pain, ROM, and WV in patients suffering from knee joint pain. Notably, the DH systems were more effective in enhancing balance and lowering pain levels in patients diagnosed with knee joint pain than ROM and WV. Despite the insignificant impact of DH systems on TKA patients in the subgroup analysis, patients with knee OA may benefit significantly from such approach. A previous study also reported that the DH systems effectively improved pain in patients with from burn injuries^26^. These findings denote the varying impacts of the DH systems on different populations. The reasons for the varied DH system performance, however, remain unclear.

The two main objectives of post-knee joint surgery rehabilitation are to relieve pain and facilitate knee function recovery, such as body weight support, balance, ROM, and WV. The deterioration of muscles, ligaments, tendons, and meniscal tissues surrounding the knee joint can adversely affect the postural balance of the patients^27^. As evidenced in the literature, the declining balance ability may lead to an increased risk of falling^22^. Therefore, the long-term functional outcomes depend on one’s ability to maintain balance. Based on the meta-analysis findings, DH systems may improve the balance ability of patients with knee joint pain more effectively than SR techniques.

For example, Ozlu et al.^10^ performed a VR game intervention on patients with knee OA five times a week for three weeks. The study showed that the balance ability of the participants in the DH group improved significantly when compared to those in the SR group. In addition, the study highlighted that DH systems may improve patients’ balance through visual and auditory stimuli^10^. Increased muscle strength and decreased pain level were also associated with improved balance, especially when DH systems were integrated^21^.

Several studies pointed out that rehabilitative training in either VR or AR offers immersive and multi-sensory effects that enable patients to experience sufficient distraction that minimizes pain sensation^28^ and enhances physical performance^29^. The concept of cognitive distraction refers to the primary working mechanism of the DH systems that regulates pain management and improves physical ability^30^. During DH systems training, patients actively participate in an immersive experience and hardly perceive stimulation beyond their field of attention, including pain. Pain sensation is generated in the thalamus region of the human brain, which is one of the most important anatomical structures that receive projections from multiple ascending pain pathways. Pain perception is also processed in the thalamus region before the information is transmitted to the corresponding cerebral cortex region. Next, the thalamus manages the pain-related factors, such as sensory discrimination (lateral pain pathway) and emotional motivation (internal pain pathway) components^31^. Another study showed that training in a virtual environment regulates the pain input intensity from the outside at the thalamus level before the cerebral cortex detects the sensory input^32^. Apart from the pain reduction mechanism via VR training, the DH systems may minimize significantly the bio-physiological parameters related to distress, particularly in declining the heart rate^33^. This exemplifies the positive impact of DH systems on pain reduction, which serves as an analgesic effect on the relevant psychological variables.

This review is limited by several apparent drawbacks. First, the nine selected articles were considered a small sample size and may not represent the entire population. Second, non-intervention as a control group is not possible in RCT for clinical population, thus we are only able to compare the impacts between DH and SR groups. Moreover, the intervention plans of both the DH and SR groups in each study were inconsistent due to the purpose of each respective study, thus hampering this present review to perform horizontal comparisons of individual included studies. Therefore, future work should include more well-designed articles to strengthen the meta-analysis findings. Finally, as it has not been found that DH intervention has a positive impact on ROM and WV, caution should be exercised when choosing DH intervention for patients who want to improve ROM and WV.

## Conclusion

The meta-analysis showed that the DH system may provide a significant impact on both pain and body function in patients with knee joint pain, in comparison to the standard rehabilitation. The DH system may improve the balance ability of patients with knee pain. Also, interventions that integrated the DH system may alleviate the pain level, particularly among OA patients. However, more RCTs with bigger sample size are required to ensure that these results are homogenous and applicable in health policies.

### Supplementary Information

Supplementary Information.

## Data Availability

The datasets used and/or analysed during the current study available from the corresponding author on reasonable request.
