# Author Correction: The impact of digital healthcare systems on pain and body function in patients with knee joint pain: a systematic review and meta-analysis

**DOI:** 10.1038/s41598-024-55342-9

**Published:** 2024-02-27

**Authors:** Longfei Guo, Shuoqi Li, Shihao Xie, Lin Bian, Shazlin Shaharudin

**Affiliations:** 1 School of Health Sciences, Universiti Sains Malaysia, Kota Bharu, Kelantan Malaysia; 2 School of Sports Science, Nantong University, Nantong, Jiangsu China; 3 School of Physical Education, Woosuk University, Jeonju, Korea

Correction to: *Scientific Reports* 10.1038/s41598-024-53853-z, published online 09 February 2024

The original version of this Article contained an error in the Abstract.

“Eight research papers were selected, which collectively involved 194 males and 279 females.”

now reads:

“Nine research papers were selected, which collectively involved 194 males and 279 females.”

The original Article has been corrected.

