# Supplementary material for: The impact of digital healthcare systems on pain and body function in patients with knee joint pain: a systematic review and meta-analysis
Source: Sci Rep. 2024 Feb 9;14:3310. doi: 10.1038/s41598-024-53853-z (PMC10853270; doi:10.1038/s41598-024-53853-z)
Supplement: Supplementary file 1 — Supplementary Information. [file 41598_2024_53853_MOESM1_ESM.docx]

**Appendix A**

Search strategy

| Databases | Search strategy | Result  (Approximately) |
| --- | --- | --- |
| Scopus | #1: Title-Abs-Key (Knee)  #2: Title-Abs-Key (Exercise or Training)  #3: Title-Abs-Key (AR or VR or "Augmented reality" or "Virtual reality")  #4: #1 and #2 and #3  Limiters - Published Date: 20030101-20230920 | 294,839  2,231,166  444,153  398 |
| Pubmed | #1: [Title/Abstract] Knee  #2: [Title/Abstract] Exercise or Training  #3: [Title/Abstract] AR or VR or "Augmented reality" or "Virtual reality"  #4: #1 and #2 and #3  Filters: Publication date from 2001/01/01 to 2023/09/20 | 177,485  823,554  90,376  153 |
| Web of Science | #1: TOPIC: (Knee)  #2: TOPIC: (Exercise or Training)  #3: TOPIC: (AR or VR or "Augmented reality" or "Virtual reality")  #4: #1 and #2 and #3  Refined by: PUBLICATION YEARS: (20230920-20030101)  Indexes=SCI-EXPANDED, SSCI, CCR-EXPANDED, | 218,528  1,855,567  292,106  316 |
| EBSCO | #1: Abstract: (Knee)  #2: Abstract: (Exercise or Training)  #3: Abstract: (AR or VR or "Augmented reality" or "Virtual reality")  #4: #1 and #2 and #3  Year: 20030101-20230920 | 281,459  1,875,242  30,449  107 |
